# Myocardial contraction fraction predicts mortality for patients with hypertrophic cardiomyopathy

**DOI:** 10.1038/s41598-020-72712-1

**Published:** 2020-10-12

**Authors:** Hang Liao, Ziqiong Wang, Liming Zhao, Xiaoping Chen, Sen He

**Affiliations:** 1 Department of Cardiology, West China Hospital, Sichuan University, 37 Guo Xue Xiang, Chengdu, 610041 Sichuan Province People’s Republic of China; 2 Department of Cardiovascular Medicine, Hospital of Chengdu Office of People’s Government of Tibetan, Chengdu, 610041 Sichuan People’s Republic of China

**Keywords:** Cardiology, Cardiovascular biology

## Abstract

The myocardial contraction fraction (MCF: stroke volume to myocardial volume) is a novel volumetric measure of left ventricular myocardial shortening. The purpose of the present study was to assess whether MCF could predict adverse outcomes for HCM patients. A retrospective cohort study of 438 HCM patients was conducted. The primary and secondary endpoints were all-cause mortality and HCM-related mortality. The association between MCF and endpoints was analysed. During a follow-up period of 1738.2 person-year, 76 patients (17.2%) reached primary endpoint and 50 patients (65.8%) reached secondary endpoint. Both all-cause mortality rate and HCM-related mortality rate decreased across MCF tertiles (24.7% vs. 17.9% vs. 9.5%, *P* trend = 0.003 for all-cause mortality; 16.4% vs. 9.7% vs. 6.1%, *P* trend = 0.021 for HCM-related mortality). Patients in the third tertile had a significantly lower risk of developing adverse outcomes than patients in the first tertile: all-cause mortality (adjusted HR: 0.26, 95% CI: 0.12–0.56, *P* = 0.001), HCM-related mortality (adjusted HR: 0.17, 95% CI: 0.07–0.42, *P* < 0.001). At 1-, 3-, and 5-year of follow-up, areas under curve were 0.699, 0.643, 0.618 for all-cause mortality and 0.749, 0.661, 0.613 for HCM-related mortality (all *P* value < 0.001), respectively. In HCM patients, MCF could independently predict all-cause mortality and HCM-related mortality, which should be considered for overall risk assessment in clinical practice.

## Introduction

Hypertrophic cardiomyopathy (HCM) is a genetically transmitted disease characterized by a broad spectrum of clinical manifestations, varying from asymptomatic and benign clinical course to adverse outcomes^1–3^. Most of the patients are presented with favorable prognosis and live a normal longevity, while for some other patients, HCM progresses along specific disease pathways and leads to mortality, including sudden cardiac death, refractory heart failure and HCM-related stroke^4^. Several factors have been identified as prognostic factors of adverse outcomes, including age, New York Heart Association (NYHA) class, family history of sudden death (FHSD), syncope, atrial fibrillation, non-sustained ventricular tachycardia, maximal wall thickness (MWT) and obstruction^5^. However, the clinical outcomes of HCM are still hard to predict due to clinical heterogeneity.

Recently, a novel volumetric index, myocardial contraction fraction (MCF), defined as ratio of stroke volume (SV) to left ventricular myocardial volume (LVMV)^6^, has been regarded as a useful predictor for cardiovascular disease events and survival in general populations^7^, patients with aortic stenosis^8^, patients with cardiac amyloidosis^9,10^ and patients with non-ischemic dilated cardiomyopathy^11^. So far, little is known about its predictive capacity for mortality in HCM patients.

Therefore, the purpose of the present study was to investigate whether MCF could predict all-cause mortality, as well as HCM-related mortality in HCM patients.

## Methods

### Study population

From December 2008 to May 2016, 499 HCM patients were identified in the study at inpatient department of West China Hospital, Sichuan University, a tertiary referral center. Diagnosis was based on the echocardiographic demonstration of an unexplained increase in wall thickness ≥ 15 mm, in the absence of abnormal load conditions^12^. Forty-one patients were excluded the study because of loss to follow up after the first evaluation. Another 20 patients without MCF values were also excluded from the study, leaving a final sample size of 438 HCM patients. Detailed information about those participants has been reported elsewhere^13^. Follow-up was conducted by clinical consultations, medical records review and telephone interviews. The study was approved by the Ethics Committee on Medical Research of West China Hospital of Sichuan University, and performed according to the principles of the Declaration of Helsinki. Due to the retrospective nature of the study, informed consent was waived.

### Echocardiographic measurement

The whole cohort underwent a standard two-dimensional transthoracic echocardiography (TTE) during baseline visits. All TTE examinations were performed following the recommendations of the American Society of Echocardiography (ASE)-European Association of Echocardiography (EAE)^14^. left ventricular mass (LVM) was calculated according to the corrected formula of the ASE-EAE as follows: LVM (g) = 0.8 × 1.04 × {[interventricular septal wall thickness (IVST) + posterior wall thickness (PWT) + left ventricular end-diastolic diameter (LVEDD)]^3^ − LVEDD^3^} + 0.6. LVMV was acquired by LVM divided by the mean density of myocardium (1.05 g/ml). Left ventricular end-diastolic volume (LVEDV) and left ventricular end-systolic volume (LVESV) were calculated from two-dimensional echo guided M-mode echocardiographic dimensions using the following formula: LVEDV (ml) = 4.5 × (LVEDD)^2^, LVESV (ml) = 3.72 × (LVESD)^2^^15^. SV (ml) = LVEDV-LVESV. Ejection fraction (EF%) was calculated as (LVEDV-LVESV)/LVEDV × 100. In the last, MCF = SV/LVMV. Left ventricular diastolic dysfunction (LVDS) was based on echocardiography diagnosis according to the guideline^16^. End-stage HCM (ESHCM) was defined by an LVEF < 50% on echocardiography during follow-up. The presence of left ventricular outflow tract obstruction was defined as a gradient > 30 mmHg at rest.

### Outcomes

The primary and secondary endpoints were all-cause mortality and HCM-related mortality, respectively. HCM-related mortality was comprised of sudden or unexpected death, death resulting from progressive hear failure, death caused by HCM-related stroke, and perioperative death due to ventricular septal myectomy.

### Statistical analysis

Continuous variables were expressed as means ± standard deviation (SD) or median (interquartile range) as appropriate. Categorical variables were expressed as frequencies (n) and percentages (%). MCF was categorized into tertiles to evaluate its influence on all-cause mortality and HCM-related mortality, with the lowest tertile (T1) serving as a referent group. Survival curves were presented as Kaplan–Meier curves, and the log-rank test was used for comparison between groups. The association between MCF and all-cause mortality and HCM-related mortality was assessed by univariate and multivariate Cox’s proportional hazard models. Baseline variables that were considered clinically relevant or that showed a univariate relationship with thromboembolism were entered into multivariate cox proportional hazards regression models. The consistency of association between MCF and endpoints in prespecified subgroups was assessed with the use of cox regression with tests for interaction. Receiver operating curve (ROC) as a function of time and the area under curve (AUC)^17^ was used to illustrate the discriminative ability of MCF for all-cause mortality and HCM-related mortality at follow-up time of 1-, 3-, 5-year. All statistical analyses were performed using Empower (R) (www.empowerstats.com, X&Y solutions, inc. Boston MA), R (https://www.R-project.org) and SPSS (SPSS Inc., Chicago, Illinois, USA). A two-sided *P* value of less than 0.05 was considered statistically significant.

## Results

### Baseline characteristics

Table 1 shows the baseline characteristics of the sample across MCF tertiles (T1: 3.5% ≤ MCF < 13.9%, T2: 13.9% ≤ MCF < 18.8%, T3:18.8% ≤ MCF < 44.3%). A total of 438 HCM patients was included in the present study. The median age at baseline was 58.0 years old (interquartile range: 46.0–67.0) and 242 patients (55.3%) were male. The average MCF was 16.7 ± 6.4. Prevalence of New York Heart Association (NYHA) III/IV, atrial fibrillation (AF) and ESHCM significantly decreased from T1 to T3. Systolic blood pressure (SBP), left ventricular diameter, EDD, ESV, SV and EF increased significantly across tertiles. In addition, there was a significant tend of decreasing left atria diameter (LA), IVS, LVPW, MWT and LVMV from T1 to T3. Other detailed information about medical histories, medications, clinical procedures and echocardiographic data are shown in Table 1.

**Table 1:** Baseline characteristics of patients with hypertrophic cardiomyopathy across MCF tertiles.

Variables	Whole cohort (n = 438)	T1 (n = 146)	T2 (n = 145)	T3 (n = 147 )	*P* trend
MCF (%)	3.5 ≤ MCF ≤ 44.3	3.5 ≤ MCF < 13.9	13.9 ≤ MCF < 18.8	18.8 ≤ MCF < 44.3	
**Basic information**					
Age (yrs)	58.0 (46.0–67.0)	57.5 (40.0–69.0)	55.8 ± 14.8	57.1 ± 13.8	0.172
Gender, male	242 (55.3%)	79 (54.1%)	75 (51.7%)	88 (59.9%)	0.355
Baseline HR (bpm)	72.0 (64.3–80.0)	75.0 (65.0–82.0)	72.0 (63.5–81.5)	72.5 (65.0–80.0)	0.347
Baseline SBP (mmHg)	120.0 (108.0–134.8)	118.0 (105.0–130.0)	120.0 (107.5–136.0)	124.0 (114.0–140.0)	0.023
Baseline DBP (mmHg)	70.0 (64.0–80.0)	70.0 (63.5–80.0)	72.0 (66.5–80.0)	71.5 (67.0–80.0)	0.254
Family history of HCM	40 (9.1%)	14 (9.6%)	16 (11.0%)	10 (6.8%)	0.443
Family history of SCD	16 (3.7%)	4 (2.7%)	10 (6.9%)	2 (1.4%)	0.032
NYHA III/IV	150 (34.2%)	66 (45.2%)	53 (36.6%)	33 (22.4%)	< 0.001
**Medical history**					
Hypertension	139 (31.7%)	43 (29.5%)	45 (31.0%)	51 (34.7%)	0.613
Diabetes	36 (8.2%)	11 (7.5%)	10 (6.9%)	15 (10.2%)	0.55
COPD	27 (6.2%)	5 (3.4%)	8 (5.5%)	14 (9.5%)	0.088
Vascular disease	32 (7.3%)	12 (8.2%)	9 (6.2%)	11 (7.5%)	0.8
Prior TE	21 (4.8%)	6 (4.1%)	5 (3.4%)	10 (6.8%)	0.363
Atrial fibrillation	76 (17.4%)	33 (22.6%)	27 (18.6%)	16 (10.9%)	0.027
**Medications/devices/procedures**					
Aspirin/clopidogrel	95 (21.7%)	33 (22.6%)	25 (17.2%)	37 (25.2%)	0.329
Warfarin	41 (9.4%)	15 (10.3%)	16 (11.0%)	10 (6.8%)	0.485
Statins	121 (27.6%)	37 (25.3%)	31 (21.4%)	53 (36.1%)	0.015
Beta-blockers	314 (71.7%)	106 (72.6%)	101 (69.7%)	107 (72.8%)	0.801
ACEI/ARB	86 (19.6%)	26 (17.8%)	22 (15.2%)	38 (25.9%)	0.057
ICD	34 (7.8%)	14 (9.6%)	16 (11.0%)	4 (2.7%)	0.018
Pacemaker	23 (5.3%)	11 (7.5%)	7 (4.8%)	5 (3.4%)	0.274
Obstruction intervention	41 (9.4%)	13 (8.9%)	16 (11.0%)	12 (8.2%)	0.683
**Echocardiographic data**	23 (5.3%)	11 (7.5%)	7 (4.8%)	5 (3.4%)	
LA (mm)	40.0 (35.0–45.0)	41.6 ± 7.5	40.0 (37.0–45.0)	38.0 (34.0–49.0)	0.021
IVS (mm)	19.0 (16.0–22.0)	23.0 (20.0–26.0)	20.0 (17.0–21.0)	16.0 (14.0–18.0)	< 0.001
LVPW (mm)	11.0 (10.0–13.0)	13.0 (11.0–16.0)	11.0 (10.0–12.0)	10.0 (9.0–11.0)	< 0.001
MWT (mm)	19.0 (16.0–22.0)	23.0 (20.0–26.0)	20.0 (17.0–21.0)	16.0 (14.0–18.0)	< 0.001
EDD (mm)	43.0 (40.0–47.0)	41.0 (37.8–45.0)	42.0 (40.0–46.0)	45.7 ± 6.3	< 0.001
ESD (mm)	26.0 (24.0–30.0)	25.0 (22.0–29.0)	26.0 (24.0–30.0)	27.0 (25.0–30.0)	0.061
EDV (mm^3^)	81.0 (68.0–99.0)	70.0 (52.0–89.0)	81.0 (69.0–98.0)	93.0 (79.0–114.0)	< 0.001
ESV (mm^3^)	26.0 (20.0–35.0)	23.0 (17.0–34.0)	26.0 (19.5–35.0)	27.0 (22.0–37.0)	0.459
EF (%)	68.0 (63.0–72.0)	67.0 (60.0–71.0)	68.0 (63.0–72.0)	70.0 (66.0–73.0)	< 0.001
LVOTO	179 (40.9%)	66 (45.2%)	61 (42.1%)	52 (35.3%)	0.217
SV (ml)	56.6 (44.0–66.0)	44.1 ± 14.1	56.7 (47.0–65.0)	68.6 (57.0–74.5)	< 0.001
LVMV (ml)	364.5 (282.7–428.8)	446.1 (347.9–513.9)	350.4 (287.7–396.0)	297.3 (240.9–332.6)	< 0.001
ESHCM	22 (5.0%)	14 (9.6%)	4 (2.8%)	4 (2.7%)	0.008
LVDS	81 (18.5%)	26 (17.8%)	30 (20.7%)	25 (17%)	0.696

*T* tertile, *MCF* myocardial contraction fraction, *HR* heart rate, *SBP* systolic blood pressure, *DBP* diastolic blood pressure, *HCM* hypertrophic cardiomyopathy, *SCD* sudden cardiac death, *NYHA* New York Heart Association, *COPD* chronic obstructive pulmonary disease, *TE* thromboembolism, *ACEI* angiotensin-converting-enzyme inhibitor, *ARB* angiotensin receptor blocker, *ICD* implantable cardioverter defibrillator, *LA* left atria, *IVST* intraventricular septal thickness, *PWT* posterior wall thickness, *MWT* maximal wall thickness, *EDD* end-diastolic diameter, *ESD* end-systolic diameter, *EDV* end-diastolic volume, *ESV* end-systolic volume, *EF* ejection fraction, *LVOTO* left ventricular outflow tract obstruction, *SV* stroke volume, *LVMV* left ventricular myocardial volume, *ESHCM* end stage HCM, *LVDS* left ventricular diastolic dysfunction.

### Outcomes

**Figure 1:**
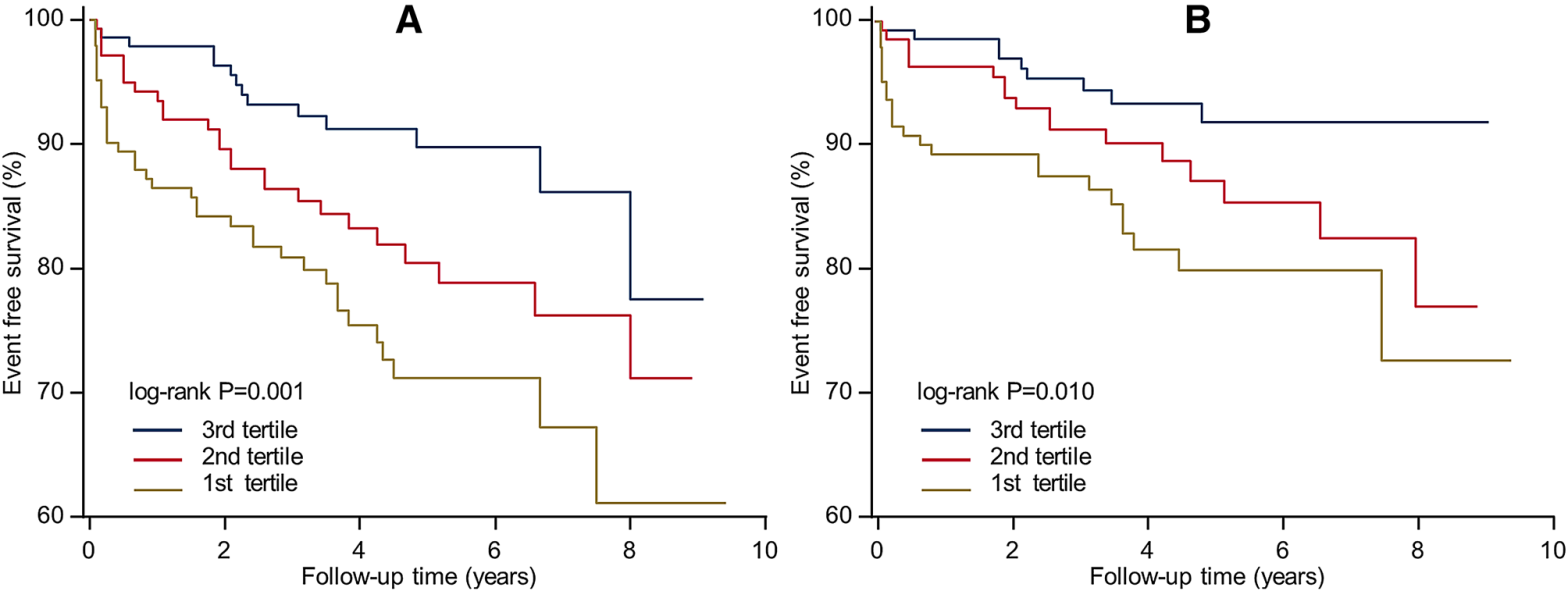
Event free survival of patients across MCF tertiles during follow-up period in HCM patients, (**A)** all-cause mortality survival curve, (**B**) HCM-related mortality curve.

During a median follow-up of 3.7 years (range 0.1–9.4) and 1738.2 person-years of observation, 76 patients had all-cause mortality with an incidence rate of 17.4%. There were 50 (65.8%) HCM-related mortality. Detail information is shown in Table 2. The number of primary and secondary endpoint across MCF tertiles is presented in Table 3. Both all-cause mortality rate and HCM-related mortality rate decreased significantly across MCF tertiles (24.7% vs. 17.9% vs. 9.5%, *P* trend = 0.003 for all-cause mortality; 16.4% vs. 9.7% vs. 6.1%, *P* trend = 0.021 for HCM-related mortality). A time-to-event analysis also indicated that patients with reduced MCF values had higher risk of all-cause mortality and HCM-related mortality (Fig. 1).

**Table 2:** Primary and secondary endpoints of the present study.

Endpoints	Data
**Primary endpoint**	
All-cause mortality	76 (100%)
**Secondary endpoint**	50 (65.8%)
Heart failure related mortality	25 (50.0%)
Stroke related mortality	9 (18.0%)
Sudden cardiac death	13 (26.0%)
Other	3 (6.0%)
**Mortality caused by other reasons**	
Cancer/car accident/GI bleeding, et al	26 (34.2%)

*GI* gastrointestinal.

**Table 3:** Multivariate Cox’s proportional hazard models for all-cause mortality and HCM-related mortality in HCM patients.

Models	All-cause mortality			HCM-related mortality		
	T1	T2	T3	T1	T2	T3
Endpoints	36	26	14	24	17	9
Mortality rate	24.7%	17.9%	9.5%	16.4%	11.7%	6.1%
Model 1	1	0.64 (0.39–1.07), 0.087	0.33 (0.18–0.60), < 0.001	1	0.63 (0.34–1.18), 0.151	0.32 (0.15–0.68), 0.003
Model 2	1	0.67 (0.40–1.11), 0.119	0.42 (0.23–0.79), 0.007	1	0.63 (0.33–1.17), 0.144	0.41 (0.19–0.90), 0.026
Model 3	1	0.60 (0.34–1.08), 0.088	0.29 (0.16–0.66), 0.003	1	0.49 (0.24–0.99), 0.045	0.20 (0.07–0.53), 0.001
Model 4	1	0.68 (0.41–1.13), 0.137	0.41 (0.22–0.76), 0.005	1	0.65 (0.35–1.21), 0.170	0.40 (0.18–0.88), 0.023
Model 5	1	0.56 (0.33–0.97), 0.037	0.26 (0.12–0.56), 0.001	1	0.46 (0.24–0.89), 0.020	0.17 (0.07–0.42), < 0.001

Model 1 adjusted for age, gender, family history of HCM and family history of SCD; model 2 adjusted for age, gender, baseline heart rate, SBP, DBP, NYHA III/IV; model 3 adjusted for age, gender, LA, MWT, EF and LVOTO; model 4 adjusted for age, gender, AF, warfarin use, syncope and ICD implantation; model 5 adjusted for age, gender, family history of SCD, syncope, NYHA III/IV, AF, MWT and LVOTO.

Abbreviations as in Tables 1 and 4.

### Survival analysis

Univariate Cox’s proportional hazard analysis revealed that patients in the third MCF tertile showed a 66% decrease of all-cause mortality and 67% decrease of HCM-related mortality when compared to that of patients in the first tertile. Other significant predictors of endpoints for HCM patients are shown in Table 2. Among them, baseline SBP and EF were protective factors. Baseline heart rate, AF, NYHA class II/IV, LA, LVPW and ESHCM were risk factors.

Five models were constructed to examine the effect of comorbidity, medication and echocardiographic parameters on the association between MCF and mortality risk. The association remained consistent and stable after adjusting different potential confounders (Table 4). After adjusting age, gender, FHSD, syncope, NYHA class III/IV, MWT and LVOTO in model 5, in comparison of the top tertile versus the bottom tertile of MCF, HRs for all-cause mortality and HCM-related mortality were 0.26 (95% CI: 0.12–0.56, *P* = 0.001) and 0.17 (95% CI: 0.07–0.42, *P* < 0.001), respectively. What’s more, no significant observations between MCF and other variables were observed during subgroup analysis (supplementary Table 1). Again, the strength and direction of the associations did not change materially.

**Table 4:** Univariate Cox’s proportional hazard analysis for all-cause mortality and HCM-related mortality in HCM patients.

Variables	Change	All-cause mortality			HCM-related mortality		
		HR	95% CI	*P* value	HR	95% CI	*P* value
Age	Per 1-year increase	1.02	1.01–1.04	0.009	–	–	–
Baseline HR	Per 1-bpm increase	1.02	1.01–1.03	0.002	1.02	1.00–1.03	0.049
Baseline SBP	Per 1-mmHg increase	0.98	0.97–0.99	0.001	0.97	0.96–0.99	< 0.001
Baseline DBP	Per 1-mmHg increase	–	–	–	0.98	0.95–1.00	0.030
COPD	Yes vs. no	3.12	1.68–5.80	< 0.001	–	–	–
AF	Yes vs. no	2.06	1.26–3.36	0.004	3.42	1.95–6.00	< 0.001
NYHA III/IV	Yes vs. no	3.12	1.98–4.92	< 0.001	2.88	1.64–5.03	< 0.001
Aspirin/clopidogrel	Yes vs. no	–	–	–	1.94	1.08–3.48	0.027
Warfarin	Yes vs. no	2.09	1.13–3.89	0.019	3.18	1.63–6.24	0.001
Beta-blockers	Yes vs. no	0.62	0.39–0.99	0.044	–	–	–
LA	Per 1 mm increase	1.03	1.01–1.06	0.027	1.05	1.02–1.09	0.003
LV	Per 1 mm increase	0.95	0.91–1.00	0.030	–	–	–
LVPW	Per 1 mm increase	1.10	1.05–1.17	< 0.001	1.03	1.03–1.18	0.004
EDD	Per 1 mm increase	0.96	0.92–1.00	0.036	–	–	–
EF	Per 1% increase	0.97	0.95–0.99	0.003	0.96	0.94–0.99	0.002
ESHCM	Yes vs. no	2.64	1.26–5.51	0.010	3.02	1.28–7.13	0.011
LVDS	Yes vs. no	0.50	0.26–0.98	0.044	–	–	–
**MCF**							
1st tertile		reference			reference		
2nd tertile		0.65	0.39–1.08	0.099	0.64	0.35–1.20	0.165
3rd tertile		0.34	0.18–0.63	0.001	0.33	0.15–0.70	0.004
*P* for trend				< 0.001			0.001

Only variables with significant association with all-cause mortality and HCM-related mortality in the univariable analysis are shown.

*HR* hazard ratio, *CI* confidence interval. For other abbreviations, see in Table 1.

### Time-dependent AUC

**Figure 2:**
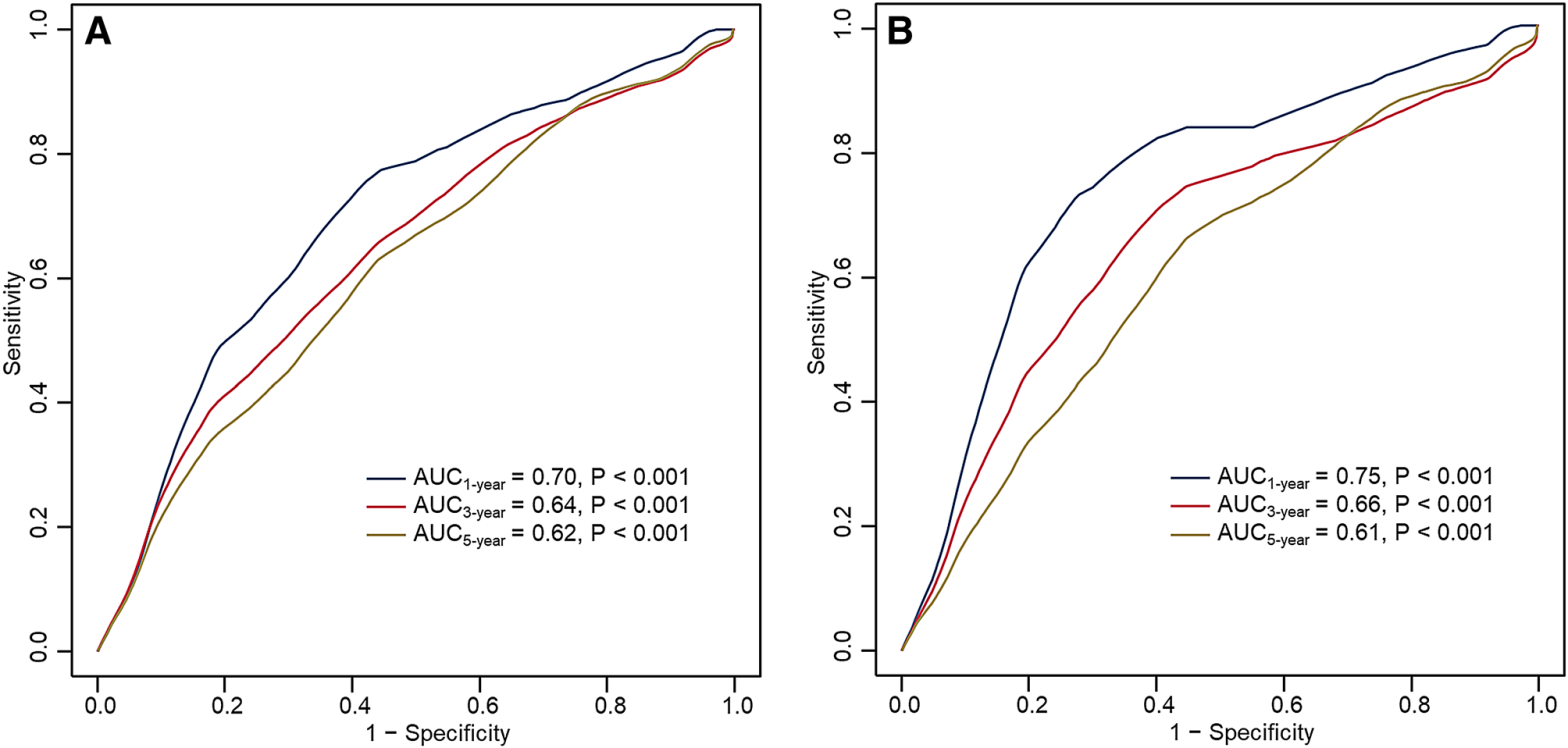
Time-dependent ROCs and AUCs of MCF for all-cause mortality (**A**) and HCM-related mortality (**B**) in HCM patients at 1-, 3-, 5-year of follow-up time.

The all-cause mortality rates were 7.1%, 12.1% and 15.8% at 1-, 3-, 5-year of follow-up, respectively. HCM-related mortality rates were 5.0%, 7.8% and 10.5%. Figure 2 depicts time-dependent ROC for MCF associated with endpoints at different follow-up time. At 1-, 3-, and 5-year, the AUCs were 0.699, 0.643 and 0.618 for all-cause mortality and 0.749, 0.661 and 0.613 for HCM-related mortality (all *P* value < 0.001). The corresponding cut-off value, youden index, sensitivity and specificity of MCF for each outcome at different timepoints are shown in supplementary Table 2.

## Discussion

In this relatively large study cohort of HCM patients, MCF was a significant and independent predictor of all-cause mortality and HCM-related mortality. The association was not changed materially after adjusting potential risk factors in multivariate Cox’s proportional hazard analysis or subgroup analysis. However, the discriminative ability showed a decreasing trend along with the extension of follow-up time.

MCF was firstly proposed by King et al. as an index of myocardial shortening which could be able to distinguish pathologic hypertensive hypertrophy and physiological hypertrophy of athletes^6^. Then a number of studies have begun to examine the relationship between MCF and cardiovascular disease (CVD) events and mortality or survival in different populations of participants. Chuang et al. analyzed data from the Framingham Heart Study cohort and concluded that patients in the lowest-quartile was 7 times more likely to develop a hard CVD event, which was consisted of cardiovascular death, myocardial infarction, stroke or new heart failure, when compared to patients in remaining quartiles^7^. In another major study cohort, the Multi-Ethnic Study of Atherosclerosis, Abdalla et al. also reported that the lowest MCF quartiles was associated with an increased risk for incident CVD (myocardial infarction, resuscitated cardiac arrest, stroke, coronary heart disease and stroke death) (HR: 2.42, 95%CI: 1.58–3.72)^18^. In patients with cardiomyopathy, including cardiac amyloidosis and non-ischemic cardiomyopathy, historical studies consistently confirmed the prognostic capacity for adverse outcomes^9–11^. Few data have evaluated the role of MCF played in patients with HCM. As far as we know, only one study ever conducted to fill this knowledge gap. In this study, Shimada et al.revealed that MCF independently predicted the composite endpoint of embolic stroke, heart transplant and cardiac death for HCM patients (HR: 0.5 per 10% increase, 95%CI: 0.28–0.90, *P* = 0.02)^19^. However, this study was largely limited by its small sample size (n = 137). The results of our study collaborated with the historical ones and mainly delineated the predictive capacity of MCF for mortality in HCM patients. The strength of results was enhanced by a relatively large number of participants (n = 438). In addition, our study is the first study to evaluate the predictive ability of MCF at different timepoints.

It is noteworthy that MCF values in our study cohort were lower than that of previous study^19^, which could be explained by the fact that this HCM cohort was enrolled at the inpatient department of a tertial referral center, patients were more exacerbated than general HCM populations. Consequently, the survival rate was apparently lower at certain timepoint when compare to the reported 1-,3-,5-year survival rates of 98.0%, 94.3%,82.2% in a recent heavy-weighted meta-analysis^4^. The predictive ability of MCF for HCM-related mortality at 1-year of follow-up was quite considerable with AUC as high as 0.75. Unfortunately, there was a decreasing trend with increasing follow-up periods which might be explained by the change of MCF itself and other risk factors and cohorts as time went by. Therefore, a dynamic evaluation based on the change of MCF may improve the predictive ability since echocardiography was a noninvasive, easily accessible routine examination for HCM patients.

Several limitations of the present study should be addressed. Firstly, this is a retrospective study with relatively small sample size and did not include a control group. Secondly, M-mode echocardiography was used for patients in our study, which might lead to LVM underestimation. However, most epidemiological reports use this imaging modality based on its technical feasibility and availability at the time when most studies were performed. So is in our retrospective study. Thirdly, patients in this study were from the inpatient department of a tertiary referral center, who tended to be sicker than the general HCM population with lower MCF values. Therefore, the strength of results may be limited when apply it to the whole HCM patients. Fourthly, it was underpowered to examine different types of cardiovascular endpoints due to small number of events, and thus we did not perform the analysis. Lastly this population was located in Chengdu, China, lack of region diversification and race comparison, the results may not be generalized to other specific patient groups or ethnicities. Large prospective studies are warranted.

## Conclusion

In conclusion, our results indicated that reduced MCF was significantly associated with increased risk for all-cause mortality and HCM-related mortality in HCM patients. MCF showed certain discriminative ability at different timepoints of follow-up, which should be considered as a useful clinical tool for mortality risk assessment among HCM patients.

## Supplementary information

Supplementary file1
