# Supplementary material for: Myocardial contraction fraction predicts mortality for patients with hypertrophic cardiomyopathy
Source: Sci Rep. 2020 Oct 12;10:17026. doi: 10.1038/s41598-020-72712-1 (PMC7552384; doi:10.1038/s41598-020-72712-1)
Supplement: Supplementary file 1 — Supplementary file1 [file 41598_2020_72712_MOESM1_ESM.docx]

Supplementary table 1. Interaction and subgroup analysis according to age, gender, NYHA class, atrial fibrillation, hypertension, LVOTO, MWT, LVDS and EF.

| Subgroups | All-cause mortality | | |  |  | HCM-related mortality | | |  |
| --- | --- | --- | --- | --- | --- | --- | --- | --- | --- |
|  | T1 | T2 | T3 | P for interaction |  | T1 | T2 | T3 | P for interaction |
| age (yrs) |  |  |  | 0.394 |  |  |  |  | 0.597 |
| < 60 | 1 | 0.46 (0.19-1.12) | 0.35 (0.11-1.08) |  |  | 1 | 0.39 (0.14-1.10) | 0.23 (0.06-0.89) |  |
| ≥ 60 | 1 | 0.59 (0.28-1.26) | 0.21 (0.07-0.64) |  |  | 1 | 0.58 (0.23-1.51) | 0.19 (0.05-0.79) |  |
| gender |  |  |  | 0.548 |  |  |  |  | 0.752 |
| male | 1 | 0.47 (0.21-1.07) | 0.24 (0.08-0.73) |  |  | 1 | 0.33 (0.12-0.92) | 0.18 (0.05-0.70) |  |
| female | 1 | 0.66 (0.30-1.43) | 0.35 (0.11-1.05) |  |  | 1 | 0.68 (0.27-1.69) | 0.26 (0.07-1.03) |  |
| NYHA class |  |  |  | 0.125 |  |  |  |  | 0.658 |
| I/II | 1 | 1.16 (0.42-3.19) | 0.76 (0.22-2.62) |  |  | 1 | 0.63 (0.20-2.03) | 0.43 (0.11-1.76) |  |
| III/IV | 1 | 0.39 (0.19-0.79) | 0.12 (0.03-0.45) |  |  | 1 | 0.41 (0.18-0.98) | 0.11 (0.02-0.53) |  |
| atrial fibrillation |  |  |  | 0.350 |  |  |  |  | 0.246 |
| yes | 1 | 0.75 (0.28-2.20) | 0.61 (0.12-3.14) |  |  | 1 | 0.83 (0.30-2.32) | 0.54 (0.10-2.83) |  |
| no | 1 | 0.50 (0.25-0.99) | 0.24 (0.10-0.61) |  |  | 1 | 0.32 (0.13-0.80) | 0.12 (0.04-0.41) |  |
| hypertension |  |  |  | 0.180 |  |  |  |  | 0.013 |
| yes | 1 | 0.53 (0.16-1.78) | 0.15 (0.02-1.02) |  |  | 1 | 0.63 (0.16-2.55) | * |  |
| no | 1 | 0.58 (0.30-1.10) | 0.34 (0.14-0.83) |  |  | 1 | 0.47 (0.21-1.06) | 0.34 (0.12-0.97) |  |
| LVOTO |  |  |  | 0.799 |  |  |  |  | 0.974 |
| yes | 1 | 0.60 (0.23-1.57) | 0.48 (0.14-1.62) |  |  | 1 | 0.53 (0.17-1.60) | 0.23 (0.05-1.06) |  |
| no | 1 | 0.66 (0.33-1.32) | 0.23 (0.09-0.64) |  |  | 1 | 0.59(0.24-1.43) | 0.26 (0.08-0.87) |  |
| MWT (mm) |  |  |  | 0.702 |  |  |  |  | 0.927 |
| < 19 | 1 | 0.81 (0.27-2.44) | 0.37 (0.11-1.18) |  |  | 1 | 0.75 (0.22-2.56) | 0.40 (0.10-1.49) |  |
| ≥ 19 | 1 | 0.67 (0.35-1.27) | 0.56 (0.17-1.85) |  |  | 1 | 0.62 (0.27-1.42) | 0.29 (0.04-1.17) |  |
| LVDS |  |  |  | 0.934 |  |  |  |  | 0.378 |
| yes | 1 | 0.21 (0.04-1.24) | 0.06 (0.004-1.22) |  |  | 1 | 0.21 (0.04-1.24) | 0.06 (0.004-1.11) |  |
| no | 1 | 0.66 (0.36-1.23) | 0.41 (0.18-0.93) |  |  | 1 | 0.67 (0.31-1.43) | 0.33 (0.12-0.93) |  |
| EF (%) |  |  |  | 0.496 |  |  |  |  | 0.167 |
| < 68 | 1 | 0.57 (0.25-1.30) | 0.27 (0.07-1.04) |  |  | 1 | 0.64 (0.23-1.75) | 0.35 (0.08-1.52) |  |
| ≥ 68 | 1 | 0.40 (0.17-0.94) | 0.22 (0.07-0.67) |  |  | 1 | 0.28 (0.10-0.78) | - 1. 0.02-0.39) |  |

*no endpoints occurred in this category.

Abbreviations as in table 1 and table 3.

Supplementary table 2. Operating characteristics of MCF to predict all-cause mortality and HCM-related mortality at different timepoints.

| Follow-up time | All-cause mortality | | | | HCM-related mortality | | | |
| --- | --- | --- | --- | --- | --- | --- | --- | --- |
|  | cut-off value | youden index | sensitivity | specificity | cut-off value | youden index | sensitivity | specificity |
| 1-year | 15.4 | 0.33 | 75.5% | 58.0% | 13.1 | 0.45 | 72.3% | 72.8% |
| 3-year | 15.6 | 0.22 | 65.3% | 56.4% | 15.0 | 0.30 | 70.3% | 60.1% |
| 5-year | 15.8 | 0.19 | 63.0% | 56.0% | 15.8 | 0.21 | 66.0% | 55.2% |

Abbreviations as in table 1.
